# Lower limbs strength variations between injured and non-injured professional soccer players

**DOI:** 10.1177/00368504231216312

**Published:** 2024-01-23

**Authors:** Francisco Martins, Cíntia França, Hugo Sarmento, Ricardo Henriques, Krzysztof Przednowek, Marcelo de Maio Nascimento, Adilson Marques, Andreas Ihle, Élvio Rúbio Gouveia

**Affiliations:** 1 Department of Physical Education and Sport, 56057 University of Madeira, Funchal, Portugal; 2 LARSYS, Interactive Technologies Institute, Funchal, Portugal; 3 Research Center in Sports Sciences, Health Sciences, and Human Development (CIDESD), Vila Real, Portugal; 4 Faculty of Sport Sciences and Physical Education, University of Coimbra, Research Unit for Sport and Physical Activity (CIDAF), Coimbra, Portugal; 5 Marítimo da Madeira—Futebol, SAD, Funchal, Portugal; 6 Institute of Physical Culture Sciences, Medical College, University of Rzeszów, Rzeszów, Poland; 7 Department of Physical Education, Federal University of Vale do São Francisco, Petrolina, Brazil; 8 CIPER, Faculty of Human Kinetics, University of Lisbon, Lisbon, Portugal; 9 ISAMB, Faculty of Medicine, University of Lisbon, Lisbon, Portugal; 10 Department of Psychology, 27212 University of Geneva, Geneva, Switzerland; 11 Center for the Interdisciplinary Study of Gerontology and Vulnerability, 27212 University of Geneva, Geneva, Switzerland; 12 Swiss National Centre of Competence in Research LIVES—Overcoming Vulnerability: Life Course Perspectives, Lausanne, Switzerland

**Keywords:** Football, sports injuries, muscular strength, isokinetic, explosive strength

## Abstract

Due to its physical demands, professional soccer is becoming a real challenge regarding players’ exposure to high injury risk. Given its tight correlation with high-intensity actions, muscular strength is a crucial physical characteristic for soccer players. Therefore, the aims of this study are (a) to compare the vertical jumping performance during the season according to the injury profile, and (b) to investigate differences in isokinetic strength performance at the beginning and the end of the season. Twenty-one male professional soccer players (age: 26.0 ± 4.1 years, height: 181.0 ± 6.9 cm, body mass: 73.7 ± 6.9 kg) were assessed in isokinetic strength (Biodex System 4 Pro Dynamometer), and Optojump Next (Microgate, Bolzano, Italy). Isokinetic strength analyses considered the peak torque scores of knee flexors and knee extensors, according to the player's preferred and non-preferred limb. The countermovement and squat jump maximum height were assessed as lower body explosive strength indicators. No significant differences were found when comparing injured and non-injured players in vertical jump and isokinetic strength assessments. However, significant results were found when comparing both groups’ initial and final evaluation in isokinetic strength assessment, with both groups significantly improving their performance. Our findings indicate that the overall lower body strength performance was not a discriminant factor between injured and non-injury players. Thus, muscular strength assessment performance increased throughout the season independently of the injury profile. Future research needs to integrate other variables related to sports injuries since they seem to result from multifactorial causes.

## Introduction

Jumping, running, and direction changes have all been considered the most crucial moves in professional soccer, leading to flagrant goal situations.^1–3^ Muscular strength significantly impacts the force-time characteristics associated with soccer performance, particularly given its close association with high-speed and high-intensity actions.^4,5^ Still, players with greater muscular strength have been associated with an inferior probability of getting injured in addition to its impact on several aspects related to sports performance.^6^ Indeed, sports agents and technical staff have been focused on evaluating players’ physical characteristics to determine their risk of injury and develop customized training programs for the season based on their profiles.^7^

The literature states that an injury is an incident that takes place during a planned practice or game and prevents the person from participating in the following practice or game.^8^ The incidence of injuries not only impacts the players’ careers but also the performance of the entire squad.^9–14^ In professional soccer, muscular injuries are the most common, accounting for 20–37% of all time-loss injuries.^8,15–18^ The causes of muscle injury in athletes are still not fully understood.^19^ There have been few studies that include professional athletes and provide the precise information on muscle injuries required for this purpose.^20^

The term “muscle strength” in the literature refers to the maximum muscular force produced by a single voluntary contraction.^21^ Contrarily, “muscle power,” often known as “torque,” refers to a force's capacity to generate rotation of a lever.^22^ In rehabilitative and athletic settings, the isokinetic dynamometer has become the gold standard for assessing static and dynamic muscle performance.^23,24^ This equipment assumes a constant angular velocity and accommodates resistance.^22^ Among the testing choices, dynamic strength may be the most frequently used indicator of a player's strength. When the muscle's tension is greater than the resistance it must overcome, a shortening is caused by the concentric muscular action (CC). These behaviors frequently appear during the positive phase of the majority of strength training sessions and may help individuals perform tasks requiring quick changes in direction and speed.^25^ The isokinetic evaluation, however, has also been characterized in the literature as a lab and analytical test that does not accurately reflect the functional components of limb movements involved in soccer play.^6^

Exercises for vertical leaping like the squat jump (SJ) and the countermovement jump (CMJ) have mostly been recommended to measure lower body strength and muscle imbalance.^4,26,27^ Although they both require specific equipment for evaluation, such as force platforms and jump applications, the SJ and CMJ are both recognized as straightforward and reliable field tests. The SJ assesses the ability to rapidly build force predominantly during the concentric movement, whereas the CMJ measures the capability to create force throughout the stretch-shortening cycle.^28^

Overall, research on strength as a measurement has been extensively addressed in the soccer literature. However, data regarding athletes’ injury profiles and how they affect the strength of their lower limbs still needs additional investigation. Therefore, the aims of this study were twofold: (a) to assess and compare the vertical jumping performance during the season according to the injury profile, and (b) to investigate differences in isokinetic strength performance at the beginning and the end of the season according to the injury profile.

## Materials and methods

### Participants

In this study, 21 professional male soccer players (age: 26.0 4.1 years, height: 181.0 6.9 cm, body mass (BM): 73.7 6.9 kg) took part. Sixteen people chose their right lower limb, whereas the remaining five chose their left lower limb. Limb preference is the preferred leg (PL) while kicking a ball, according to one definition.^29^ During the 2021–2022 season, all competitors played in the First Portuguese League.

The research team's qualified personnel completed each evaluation in a physical performance lab. The Ethics Committee of the Faculty of Human Kinetics, CEIFMH N° 34/2021, authorized the techniques that were used. All participants gave their informed permission after the inquiry was performed in accordance with the principles of the Declaration of Helsinki.

### Injury report

The Union of European Football Associations (UEFA) made recommendations for epidemiological studies that were followed in this study. An injury was defined as an occurrence during a planned practice or game that prevented the participant from participating in the subsequent practice or game.^8^ The clinical section kept daily injury reports throughout the season, covering training and competitive events. Type, zone, exposure, incidence, severity, and occurrence of injuries were the factors evaluated. Additionally, the minute of the incident was recorded if an injury happened during a game. All of the injuries suffered throughout the season were documented. According to the severity of the injuries, two groups were created: wounded and non-injured.

The type and zone of the injury are two complementing factors that help determine which region of the body had structural and/or functional changes as a result of the injury's contraction. The number of days from the player's halt till they may return to fieldwork with the clinical department's approval determines the severity of the injury. Last but not least, a weekly report on injuries was provided, including whether they happened during a training or official match session. Players who sustained injuries at the conclusion of the athletic season were monitored until they had fully recovered.

### Vertical jump

The maximal heights of the SJ and CMJ were used to assess the ability to leap vertically.^30^ Participants completed four data-collecting trials spaced out by 30 s. The Optojump Next (Microgate, Bolzano, Italy) system of analysis and measurement was used to get the data. Despite evidence supporting a 1-min passive break in between leaps to promote muscle recovery,^31^ the literature is divided on the subject. Due to time constraints in our study, particularly in the professional team, we took into consideration a 30 s rest break between each jump performance. Shorter recovery durations, such as 20 s^32^ and 30 s^33^ between each repetition of the CMJ, have also been reported in several investigations. During testing, participants were urged to leap as high as possible. Each participant was given three experimental trials to confirm proper execution after the protocol's explanation.

Participants stood at the start of the CMJ with their feet hip-width to shoulder-width apart. From here, participants descended into almost 90 degrees of knee flexion before executing a maximum effort vertical leap. The hands stayed on the hips the entire time to prevent the effects of arm swing. If excessive knee flexion or hand removal from the hips occurred throughout the trial, it was repeated. After every leap, the participants repositioned themselves.^34^ For the SJ, the contestants first squatted with their knees bent around 90 degrees, then executed a vertical jump with all of their might. If a hip dipping motion was seen, the trial was repeated. After every leap, the participants repositioned themselves.^34^ The assessments of vertical jumps were carried out throughout the 2021/2022 season, every 2 weeks, from the pre-season period (in the last week of June 2021) until the week referring to the last game of the sporting season analyzed (until the mid-month of May 2022).

### Isokinetic strength assessment

Isokinetic strength measurements were performed on the hamstrings and quadriceps muscles using the Biodex System 4 Pro Dynamometer (Shirley, NY, USA). The isokinetic strength of knee extensors (KEs) and knee flexors (KFs) from the PL and non-preferred leg (NPL) was recorded at an angular velocity of 60°/s. Prior to data collection, a 5-min warm-up in a reclining bicycle (Technogym Xt Pro 600 Recline, Cesena, Italy) was performed with an effort varying from levels 4 and 5, and at a cadence ranging between 50 and 60 rotations per minute. Following the manufacturer's instructions, participants were placed in the dynamometer and instructed to assume a standard hip flexion angle of 85 degrees from the anatomical position. The lateral epicondyle of the knee served as the alignment point for the dynamometer's lever arm, which was belt-stabilized along with the trunk and the leg and thigh being tested. Participants were required to extend their knees to their fullest extent in order to determine the range of motion. Participants were then instructed to stretch their knees to a 90-degree angle. Individual calibration for gravity correction was performed at 30° of knee flexion as recommended previously.^35^ During testing, participants were asked to keep their arms crossed with the hand on the opposite shoulder holding the belts,^36^ and encouragement and enthusiastic verbal support were given throughout the tests. Three repetition trials were given before testing to ensure correct execution.^37^ After, five repetitions of concentric contraction efforts of knee flexion and knee extension were performed at 60°/s, with a 60 s interval. The analysis included the peak torque (PT) of KFs and KEs in the PL and NPL. PT is described in the manufacturers’ manual.^38^ The initial assessment of isokinetic strength was carried out before the pre-season period (in the last week of June 2021) and the final assessment was performed in the last week of league stoppage (in the last week of March 2022).

### Statistics

Descriptive statistics are presented as frequencies, proportions (%), and means ± standard deviations. The injury burden was estimated as the number of days of absence per number of injuries. Absolute values present the frequency of muscular injuries by body zone. A Mann–Whitney U test explored differences between groups in chronological age (CA), body composition variables, and isokinetic strength assessments. A mixed ANOVA (between groups within subjects) was conducted to assess the impact of injury and time on players’ vertical jump performance (CMJ and SJ) across different moments of the season. The Wilcoxon Signed Rank Test verified differences in isokinetic strength variables between the first and second evaluation moments. The normality and homogeneity of variance were respected. All the analyses were performed using the IBM SPSS Statistics software 28.0 (SPSS Inc., Chicago, IL, USA). The significance level was set at 5%.

## Results

### Injured and non-injured players’ profiles

Table 1 shows comparison findings across groups during their initial evaluation at the start of the season, as well as descriptive data for CA and body composition. Between injured and non-injured athletes, there were no appreciable statistical differences in the non-modifiable and modifiable factors.

**Table 1.** Descriptive statistics for CA and body composition and comparison results between groups.

Median (range)				Mann–Whitney *U* Test	
Variables	All players (n = 21)	Injured (n = 6)	Non-injured (n = 15)	U	*p*
CA (years)	25.6 (19.6–34.6)	25.4 (20.4–31.5)	25.6 (19.6–34.6)	44.000	0.970
Height (cm)	181 (166–192)	180.5 (171–189)	181 (166–192)	47.000	0.910
BM (kg)	77.7 (62.6–88.3)	76.9 (66.3–85.6)	77.7 (62.6–88.3)	47.000	0.910
BF (%)	11.8 (7.7–20)	11.6 (7.7–15)	12 (8.3–20)	44.000	0.970
FFM (kg)	67.7 (52.7–79)	67.3 (58.9–79)	67.7 (52.7–75.9)	50.000	0.733

CA: chronological age; BM: body mass; BF%: body fat percentage; FFM: fat-free mass, 95% *CI:* 95% confidence interval.

### Muscular injuries characterization

Of a total of 19 injuries that were contracted by these players across the 2021/2022 season, six of them were muscular injuries. On average, a player sustained 0.28 muscular injuries throughout the season. So, the prevalence of muscular injuries was roughly 32% throughout the season for the 21 players that composed this sample. On average, in every three injuries, one affected the muscular structure of these professional soccer players.

Table 2 shows the number of muscular injuries according to the body zone. The quadriceps and adductors were the most affected zones, with two injuries each. Moreover, adductor injuries cost a mean of 38 days off, leading to the highest number of days of absence from matches and training sessions by a professional player. Overall, none of the players’ time off was considered minimal, demonstrating that the impact of muscular injuries is indeed of concern in professional soccer.

**Table 2.** Incidence of muscular injuries according to the body zone.

Body zone	n	Total absence, d	Severity^a^	Injury burden^b^	Missed trainings	Missed matches
Quadriceps	2	18	Moderate	9	13	3
Hamstrings	1	34	Severe	34	24	5
Adductor	2	76	Severe	38	27	6
Abdominal	1	6	Mild	6	4	1
Total	6	134	Moderate	22.3	96	19

a Average days missed by players due to an injury, *minimal:* 1–3 days, *mild:* 4–7 days, *moderate:* 8–28 days, *severe* +28 days.

b Injury burden is expressed as the number of days of absence/number of injuries.

### Lower body strength assessment

**Figure 1.**
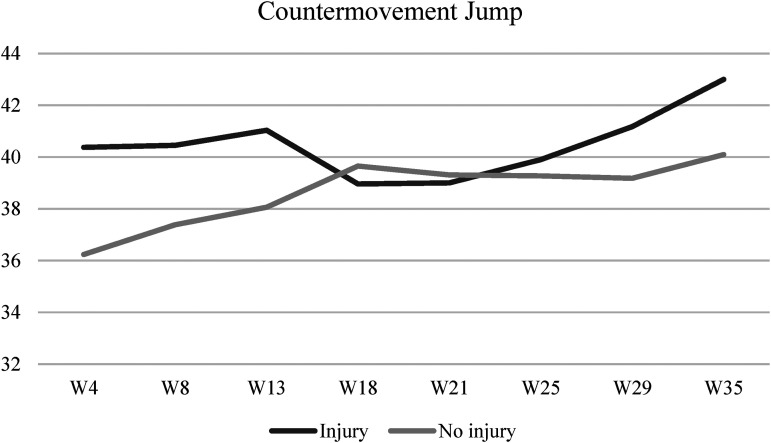
CMJ's performance across eight-time points during the season according to the injury profile. CMJ: countermovement jump.

**Figure 2.**
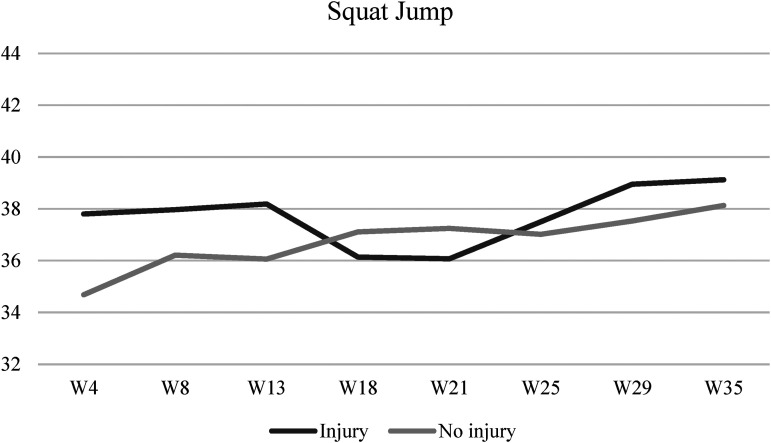
SJ's performance across eight-time points during the season according to the injury profile. SJ: squat jump.

Figure 1 illustrates the CMJ performance across eight-time points during the season, considering the injury profile. The same analysis is presented for SJ in Figure 2. No significant interaction was found between the injury profile and time for vertical jumping (CMJ: Wilks’ Lambda = 0.60, F (7,13) = 1.25, *p* = 0.35, η^2 ^= 0.40; SJ: Wilks’ Lambda = 0.62, F (7,13) = 1.15, *p* = 0.39, η^2 ^= 0.38).

Table 3 displays descriptive statistics and comparisons between groups for isokinetic strength variables. Generally, there were no statistical differences with significance between injured and non-injured players when performing with the PL and the NPL.

**Table 3.** Descriptive statistics for isokinetic strength assessment and results of Mann–Whitney U test.

Median (range)				Mann–Whitney *U* Test		
Variables	All players (n = 21)	Injured (n = 6)	Non-injured (n = 15)	U	*p*	*r*
Preferred limb						
KE PT (1^st^)	199.5 (137–293)	183.1 (153–289)	215.9 (137–293)	36.000	0.519	0.153
KE PT (2^nd^)	249.3 (117–329)	266.7 (215–329)	247.9 (117–320)	51.500	0.622	0.111
KF PT (1^st^)	123.8 (55–182)	112.0 (94–161)	123.8 (55.2–182.5)	37.500	0.850	0.127
KF PT (2^nd^)	145.0 (83–190)	151.7 (113–190)	145.0 (83–182)	51.000	0.910	0.101
Non-preferred limb						
KE PT (1^st^)	215.3 (126–297)	206.1 (160–293)	215.3 (126–297)	69.000	0.569	0.051
KE PT (2^nd^)	256.8 (149–331)	257.5 (149–301)	256.8 (176–331)	43.000	0.677	0.034
KF PT (1^st^)	111.6 (66–161)	114.2 (104–157)	111.6 (66–161)	70.000	0.791	0.068
KF PT (2^nd^)	152.0 (93–186)	160.2 (138–186)	149.2 (93–178)	85.500	0.132	0.331

*KE:* knee extensors; *KF:* knee flexors; *PT:* peak torque; 1^st^ first moment of evaluation, 2^nd^ second moment of evaluation, 95% *CI* 95% confidence interval.

**Figure 3.**
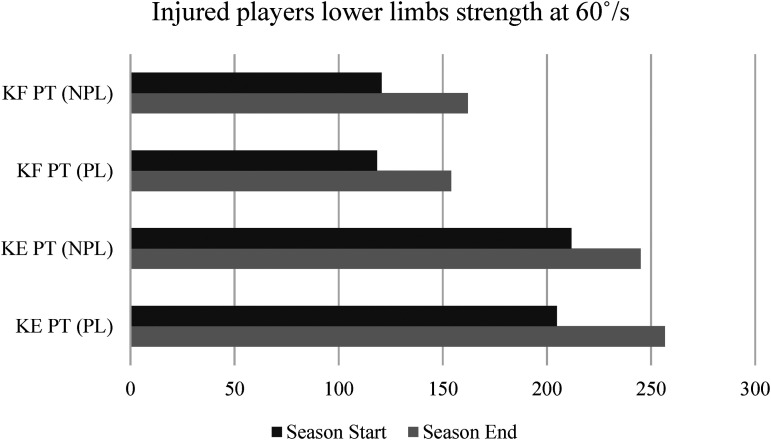
Isokinetic strength assessment among injured players between the beginning and the end of the season.

**Figure 4.**
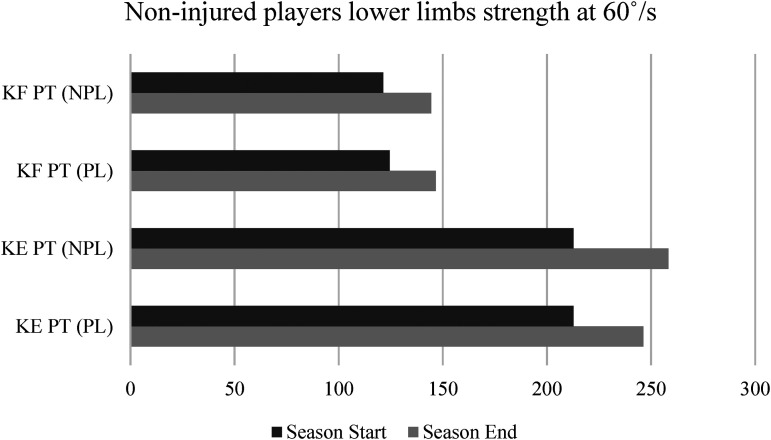
Isokinetic strength assessment among non-injured players between the beginning and the end of the season.

Figure 3 (injured players) and Figure 4 (non-injured players) present the differences between the beginning and the end of the season in isokinetic strength assessment at 60°/s for the PL and NPL. In the injured group, the results indicate a significant increase from the season start to the season end in KE PT (z = –1.992, *p* ≤ 0.05, large effect size) and KF PT (z = –1.922, *p* ≤ 0.05, large effect size) while performing with the PL. While performing with the NPL, a significant increase was only found for KF PT (z = –2.201, *p* ≤ 0.05, large effect size). Although KE PT has also increased between the two time points, the results were not statistically significant (z = –1.572, *p* = 0.12).

In the non-injured group, significant statistical differences were observed in all variables analyzed. KE PT (z = –1.931, *p* ≤ 0.05, medium effect size) and KF PT (z = –2.606, *p* ≤ 0.01, large effect size) increased substantially for the PL. Additionally, significant increases were detected for KE PT (z = –2556, *p* ≤ 0.01, medium effect size) and KF PT (z = –2.272, *p* ≤ 0.05, medium effect size) in the performance with the NPL.

## Discussion

This study aimed to assess and compare the vertical jumping performance during the season according to players’ injury profile and to analyze differences in isokinetic strength performance at the beginning and the end of the season according to players’ injury profile. Consequently, analysis and characterization of the muscular injuries of the sample were also carried out to profile the groups of injured players and the group of non-injured players. This study has shown no significant differences between both groups in terms of age, height, and baseline parameters of body composition. This may be related to the fact that they all belong to the same professional football team, sharing daily habits that make them very homogeneous in their physical condition.^19^

Although no significant differences were observed, particularly in body composition parameters in our study, it remains essential to monitor these variables throughout a sports season. Literature has reinforced body composition variables such as BM, body fat percentage (BF%), and indirectly fat-free mass as crucial conditions to enhance players’ physical capacities such as jumping, sprinting, and changes of direction.^19,39–42^ Thus, technical teams will have the opportunity to control the body parameters of their players and adapt aspects such as field and gym work and nutrition. In this group of players, a weekly monitoring process was carried out throughout the season so that the same care could be taken throughout the sporting season analyzed in this study.

In terms of muscular injury characterization in this study, the prevalence of muscular injuries was approximately 32% throughout the analyzed season. Several studies in the literature have reached the same results, as at the level of professional football, it seems that muscle injuries affect about 20–37% of total injuries.^8,15,16,18^ Moreover, several studies have stated that muscular injuries are the most common injuries a professional football player has to deal with throughout his sporting career.^9,20,43^ On the other hand, the average number of muscular injuries per athlete was 0.28 over the 2021/2022 season. Overall, other studies in the literature showed higher average values of muscular injuries over a sports season.^18,20^ A study conducted over three seasons with 227 young professional soccer players aged 16.8 ± 3.1 years reported an average of 0.92 muscle injuries per player.^17^ Also, a longitudinal study across nine sportive seasons with a sample of 2299 professional soccer players, described 0.6 muscular injuries per player.^20^ Thus, the results found in our study are encouraging for the type of work that the technical staff of this professional football team has been developing in terms of muscle strengthening. A preventative injury program, mostly focused on mobility, myofascial, proprioceptive, plyometric, eccentric strength, bilateral and unilateral strength, and isometric and dynamic core exercises, was applied to the professional soccer group that was the subject of our study. The athletes completed these workouts, which rotated between the field and the gym, in two sets with four to eight repetitions each, lasting 15 to 25 min. In addition to the type of muscular reinforcement work being linked to the low average number of muscular injuries, in comparison with the literature, biological, psychological, and physiological variables may also be at the base of the differences presented by this group of players. Regardless of this perspective, this key information could make sports agents and coaches more aware of the need to monitor players’ injuries and put preventative measures in place during the season.^19^

Concerning muscular injury frequencies, the players in this group suffered six muscular injuries during the season. The most affected body zones were the quadriceps and adductors, with the hamstrings and abdominal also being affected. Compared to data reported by a 9-year study with the participation of 24 clubs selected by UEFA and a total of 2, the areas most affected by muscle injuries were effectively the hamstrings, quadriceps, and adductors.^20^ The main difference between studies is related to the fact that in our study, players stayed out for an average of 3 weeks due to muscle injuries, a relatively high figure compared to the 2 weeks reported by the aforementioned study.^20^

In terms of explosive strength, both groups improved their CMJ and SJ performance when comparing the season's first and last evaluations. It was thus possible to observe that the group of injured athletes presented higher values of CMJ and SJ at the beginning and end of the season, and the group of athletes without muscular injuries only presented better results approximately in the middle of the sportive season. A study made across one sportive season with the participation of 36 male professional soccer players concluded that for every additional 1 centimeter in height attained during the SJ, the risk of suffering an injury rises by 1.47.^44^ These results are in line with earlier research in that explosive power significantly contributes to players having more chance of sustaining an injury.^45^ On the contrary, a recent study across a season that includes 81 young elite team-sports athletes has concluded that athletes with less vertical jump capacity have greater chances of getting injured.^46^ A previous systematic review on the topic showed conflicting results on the link between muscular strength and the risk of injury.^47^

Regarding the isokinetic strength evaluation, significant improvements were noticed when comparing the performances of KF PT and KE PT, independently of the lower limb being dominant or non-dominant and independently of the players’ injury profile. These results highlight that regardless of the player's profile in terms of sports injuries, all players tend to increase their lower limb strength when comparing the initial and final evaluations of a sports season. Thus, the injury prevention work already carried out by the technical team of this team shows significant results in increasing the players’ muscular strength in the lower limbs. A study conducted on 20 sub-elite football players applying an injury prevention training program concluded, through analysis of isokinetic data, that there was a substantial improvement in the strength of the players’ lower limbs after participation in this prevention program.^48^ Indeed, some studies have already reinforced the fact that the isokinetic dynamometric systems have been used for specialized strength training, post-surgical musculoskeletal process rehabilitation, prevention of muscle imbalances that increase the risk of muscle injuries, evaluation of lower extremity muscle strength and power in soccer players, and many other purposes.^49,50^ Besides, this professional football team presented initial and final PT values on knee extension in the preferred lower limb of 199.5 N/m and 249.3 N/m, respectively.

Regarding initial and final values of the PT on the flexors of the preferred lower limb, they were 123.8 N/m and 145.0 N/m, respectively. In other studies performed on professional football teams, the PT values for the KEs vary between 237.3 N/m and 283.4 N/m and for the KFs between 174.4 N/m and 177.8 N/m on 60°/s.^51,52^ Once more, those facts ended up approaching those in the parameters of other studies at the end of the season, embellishing the preventive work and muscle strengthening carried out.

This study has some limitations that must be noted. The study's primary shortcomings are its small sample size and lack of players’ information on previous injuries. Nevertheless, football staff working in professional soccer clubs may benefit from these practical implications regarding muscular injuries and their repercussions throughout a sportive season. Muscle injuries have come to be felt as the most common injuries throughout the career of a professional footballer. Thus, this is a rising subject, and its study is crucial in preventing and recovering from sports injuries. Ultimately, the main goal will be for this information to help technical staff and their players improve injury prevention and, consequently, the individual and collective performances of those involved.^20,53^

## Conclusions

Due to the homogeneity of this professional soccer team, statistical differences were not found in the lower limb strength assessments through the analyzed sports season when concerning players’ injury profiles. The main results of this study showed that, independently of the injury profile, professional football players seemed to increase their lower limb strength throughout the season. Moreover, this study's prevalence of muscular injuries is below what is frequently reported by the literature. These two main results could be good indicators of the preventive work the technical team carefully implemented. This investigation reinforces that muscular strengthening work and injury prevention is central to the availability of players to play and should continue to be studied in the context of high-performance sport. The results of the present study may help technical teams, coaches, and their players to identify variables and outcomes that may bring players closer to injury. Furthermore, using simple and precise indirect (optojump next) and direct instruments (isokinetic dynamometer) of strength quantification facilitates the communication between theory and practice.
